# CMT2Q-causing mutation in the *Dhtkd1* gene lead to sensory defects, mitochondrial accumulation and altered metabolism in a knock-in mouse model

**DOI:** 10.1186/s40478-020-00901-0

**Published:** 2020-03-13

**Authors:** Chun-jie Luan, Wenting Guo, Lei Chen, Xi-wei Wei, Yimin He, Yan Chen, Su-ying Dang, Robert Prior, Xihua Li, Ying Kuang, Zhu-gang Wang, Ludo Van Den Bosch, Ming-min Gu

**Affiliations:** 1 Department of Medical Genetics, E-Institutes of Shanghai Universities, Shanghai Jiao Tong University School of Medicine (SJTUSM), 280 South Chongqing Road, Shanghai, 200025 People’s Republic of China; 2 Department of Neurosciences, Experimental Neurology and Leuven Brain Institute (LBI), KU Leuven, University of Leuven, Leuven, Belgium; 3 VIB, Center for Brain & Disease Research, Laboratory of Neurobiology, Leuven, Belgium; 4 Department of Neurology, Children’s Hospital of Fudan University, Shanghai, China; 5 Shanghai Research Centre for Model Organisms, Shanghai, China; 6 Research Center for Experimental Medicine, Rui-Jin Hospital at SJTUSM, Shanghai, China

## Abstract

Charcot-Marie-Tooth disease (CMT) is a group of inherited neurological disorders of the peripheral nervous system. CMT is subdivided into two main types: a demyelinating form, known as CMT1, and an axonal form, known as CMT2. Nearly 30 genes have been identified as a cause of CMT2. One of these is the ‘dehydrogenase E1 and transketolase domain containing 1’ (*DHTKD1*) gene. We previously demonstrated that a nonsense mutation [c.1455 T > G (p.Y485*)] in exon 8 of *DHTKD1* is one of the disease-causing mutations in CMT2Q (MIM 615025). The aim of the current study was to investigate whether human disease-causing mutations in the *Dhtkd1* gene cause CMT2Q phenotypes in a mouse model in order to investigate the physiological function and pathogenic mechanisms associated with mutations in the *Dhtkd1* gene in vivo. Therefore, we generated a knock-in mouse model with the *Dhtkd1*^Y486*^ point mutation. We observed that the *Dhtkd1* expression level in sciatic nerve of knock-in mice was significantly lower than in wild-type mice. Moreover, a histopathological phenotype was observed, reminiscent of a peripheral neuropathy, including reduced large axon diameter and abnormal myelination in peripheral nerves. The knock-in mice also displayed clear sensory defects, while no abnormalities in the motor performance were observed. In addition, accumulation of mitochondria and an elevated energy metabolic state was observed in the knock-in mice. Taken together, our study indicates that the *Dhtkd1*^Y486*^ knock-in mice partially recapitulate the clinical phenotypes of CMT2Q patients and we hypothesize that there might be a compensatory effect from the elevated metabolic state in the knock-in mice that enables them to maintain their normal locomotor function.

## Introduction

The name Charcot-Marie-Tooth disease (CMT) originates from the French neurologists Jean Martin *Charcot* and Pierre *Marie*, as well as the English neurologist Howard *Tooth*, who first described the disease in 1886 [1]. The clinical name for CMT is ‘hereditary motor and sensory neuropathy’ and it is one of the most common inherited disorders of the peripheral nervous system with a prevalence of 1 in 2500 individuals [2]. Clinically, CMT is characterized by progressive muscular and sensory defects starting at the distal extremities with chronic atrophy and weakness [3]. Based on the upper limb motor nerve conduction velocities (MNCVs) (median or ulnar nerve), CMT is classified into two main forms: a demyelinating (CMT1) and an axonal form (CMT2). Furthermore, accompanying with a family history of the neuropathy, CMT1 is defined when MNCVs are ≤35 m/s and CMT2 is defined when MNCVs are ≥45 m/s. Intermediate forms of CMT also exist, which are characterized by MNCVs between 35 and 45 m/s. Genetically, CMT is a heterogeneous group of diseases with more than 80 disease-associated genes identified to date [4]. At least 29 unique genes have been associated with CMT2, which represents 25 to 30% of all CMT cases (http://www.molgen.ua.ac.be/CMTMutations/default.cfm). Several CMT2-causing genes are mitochondria- specific genes, and/or lead to defects in mitochondrial function, axonal transport, and/or in the axonal cytoskeleton [2, 5, 6]. As a consequence, mitochondrial dysfunction has been suggested as a critical pathological component of the disease mechanisms of CMT2. Moreover, these deficits in mitochondrial function and transport could dramatically affect the peripheral nerves by depriving the distal axon of an important energy source [6–8].

In 2012, we identified a new gene responsible for an autosomal dominant axonal form of CMT in a five-generation family with CMT2 which had been classified as CMT2Q [MIM 615025] [9]. We discovered a nonsense mutation, c.1455 T > G (p.Y485*), in exon 8 of *DHTKD1*. The *DHTKD1* gene encodes a ‘dehydrogenase E1 and transketolase domain-containing 1’ protein that is hypothesized to interact with proteins involved in mitochondrial energy production [9–11]. We observed that DHTKD1 mRNA expression levels in peripheral blood of CMT2Q patients decreased to half compared with unaffected individuals [9]. Similarly, in vitro studies showed that mutant mRNA and truncated DHTKD1 were significantly decreased by nonsense-mediated mRNA decay (NMD) [9]. In addition, *DHTKD1* silencing was found to lead to impaired energy production. These data demonstrated that CMT2Q could be caused by the nonsense mutation in *DHTKD1* [9]. Subsequently, we reported a strong correlation of DHTKD1 expression levels with ATP production, revealing that DHTKD1 plays a critical role in energy production through mitochondrial biogenesis and function maintenance [9, 12]. However, the pathogenic mechanisms underlying axonal CMT2Q remain elusive.

In an attempt to recapitulate the phenotype of CMT2Q, as well as to investigate the *Dhtkd1*^Y486*^ point mutation in vivo, we generated a *Dhtkd1*^Y486*^ knock-in mouse model. We observed that the knock-in mice showed some features comparable to human CMT2Q. These include lower Dhtkd1 level, reduced axon diameter, abnormal myelination, and sensory defects. While these mice did not show any abnormalities in their locomotor performance, we found accumulation of mitochondria and elevated energy metabolism in *Dhtkd1*^Y486*/Y486*^ mice. This is contradictory to the low energy state in our previous in vitro study. Therefore, we propose that a compensatory mechanism exists in the peripheral nervous system of the *Dhtkd1*^Y486*^ knock-in mice through an elevation of their metabolic state.

## Results

### Development of a *Dhtkd1*^Y486*^ knock-in mouse model that mimics NMD observed in CMT2Q patients

**Fig. 1:**
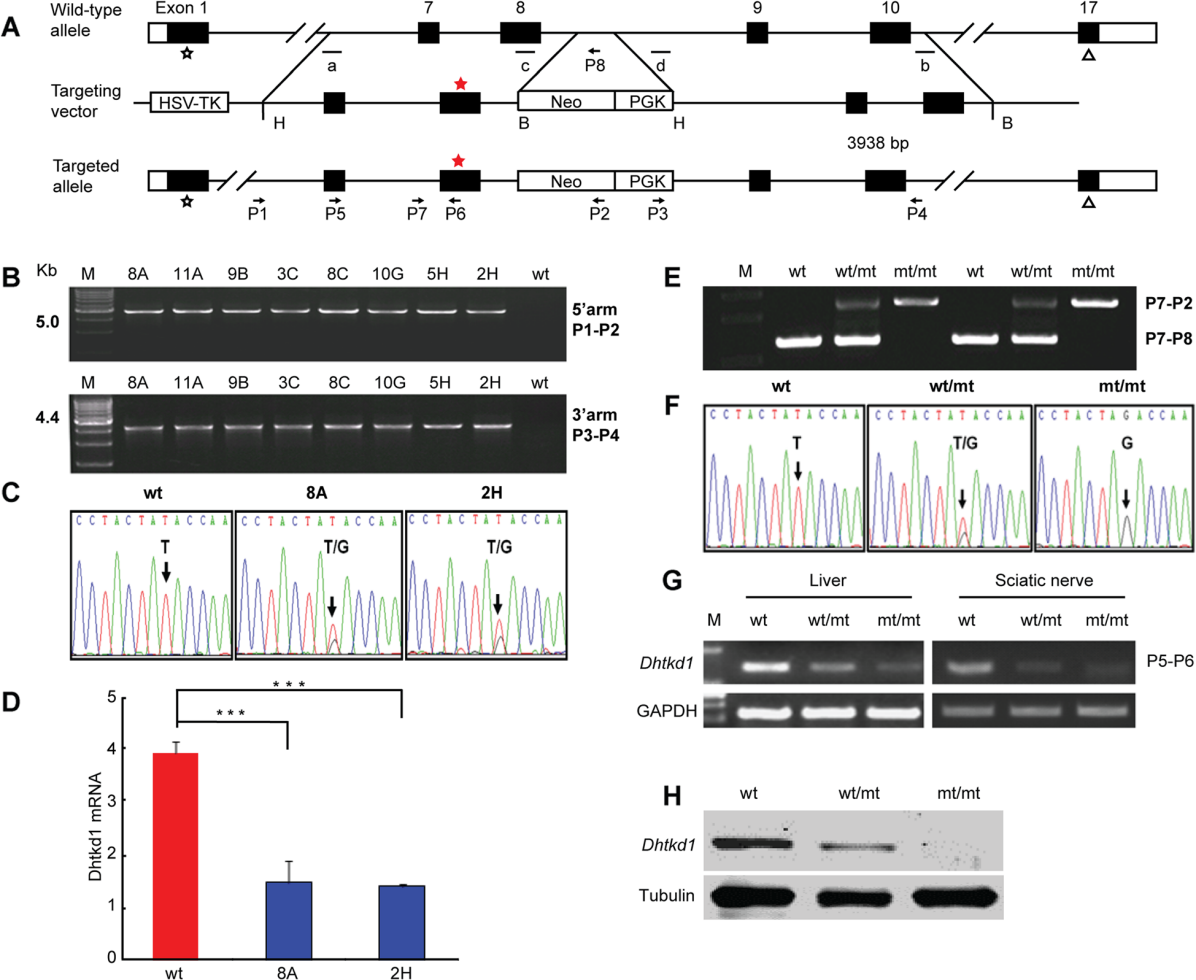
Strategy used for establishing the *Dhtkd1*^Y486*^ knock-in mouse model and the expression profile of Dhtkd1. **a** Schematic illustration of the strategy used to generate the *Dhtkd1*^Y486*^ knock-in mice. The first schematic line indicates the *Dhtkd1* allele in the wild-type (wt) mouse. Exons and introns are represented by boxes and horizontal lines, respectively. The coding regions are shown in black. Positions of the start codon and the stop codon are indicated by star and delta sign, respectively. Homologous recombination results in the point mutation and the replacement of a small part of the intron between exon 8 and exon 9. The second line shows the targeting construct. PGK-Neo cassettes are used for positive selections. The third line shows the targeting alleles. P1-P8 indicates the primers used for genotyping. **b** Identification of positive ES cell clones by PCR amplification with primers indicated in the Fig. M = DNA molecular ladder. **c** Sequencing of positive ES cell clones confirms the mutation, with the DNA base variations indicated in the figure by arrows. **d** Relative *Dhtkd1* mRNA level, normalized to *Gapdh*, from ES cells showing a significant decrease in *Dhtkd1* mRNA level in mutant lines. **e** Genotyping showed the *Dhtkd1* in wt, heterozygous (wt/mt), and homozygous (mt/mt) mice with respect to primers P7-P2 and P7-P8. **f** Sequencing of genomic DNA from mouse tails confirms the mutation. Sequence DNA base variations are indicated by arrows. **g** RT-PCR of *Dhtkd1* starting from total RNA from liver and sciatic nerve in wt, wt/mt, and mt/mt mice. *Gapdh* was used as an internal control. **h** Western blot with kidney tissue of wt, wt/mt, and mt/mt mice. One of three independent experiments in triplicate is shown. ***p < 0.001

To generate the *Dhtkd1*^Y486*^ knock-in mice, we constructed a targeting vector using the Red/ET cloning method and used homologous recombination in the embryonic stem (ES) cells to obtain the *Dhtkd1*^Y486*^ mutation in mice [13]. The translated region of the mouse *Dhtkd1* gene includes 17 exons spanning approximately 2766 bp on mouse chromosome 2. The genomic clones were isolated from a 129/SvJ mouse bacterial artificial chromosome (BAC) genomic library that contained genomic fragments encompassing the whole mouse *Dhtkd1* gene. The targeting vector for the *Dhtkd1*^Y486*^ mutation was constructed using a plasmid encompassing exons 7–10 of the *Dhtkd1* gene. Two partially complimentary oligonucleotides were used to introduce the Y486* point mutation into the targeting vector. Part of the intron between exon 8 and exon 9 was replaced by PGK-Neo (Fig. 1a). Nine ES cell clones were identified as being positive by polymerase chain reaction (PCR) using primers P1 and P2 directed to the 5′ arm and primers P3 and P4 to the 3′ arm (Fig. 1b). Two independent ES cell clones (8A and 2H) that contain heterozygous *Dhtkd1*^Y486*^ mutation were confirmed by Sanger sequencing (Fig. 1c). Interestingly, we observed that the *Dhtkd1* mRNA reduced by over 50% in heterozygous ES cell clones compared to wild-type (wt) ES cells (Fig. 1d). Following this, the two confirmed ES cell clones were injected into pseudo-pregnant females to generate chimeras. The germline transmission of the *Dhtkd1*^Y486*^ mutation was confirmed by PCR analysis on genomic DNA. Subsequently, male chimeras were crossed with C57BL/6 J females to establish strains with a mixed genetic background (C57/129) which were heterozygous for the mutant allele. *Dhtkd1*^Y486*/+^ mice were bred to generate *Dhtkd1*^Y486*/Y486*^, *Dhtkd1*^Y486*/+^ and wt (*Dhtkd1*^+/+^) littermates. Genotypes were determined by PCR using specific primers targeting either the mutant or wt allele (P7 and P2 to the *Dhtkd1*^Y486*^ allele and P7 and P8 to the wt allele) (Fig. 1e). The presence of the Y486* point mutation was further validated by Sanger sequencing (Fig. 1f). The cumulative genotype ratios (*Dhtkd1*^Y486*/Y486*^, *Dhtkd1*^Y486*/+^ and wt) for all mice are in line with the Mendelian ratios (1:2:1) (Sup. Table 1). Both *Dhtkd1*^Y486*/Y486*^ and *Dhtkd1*^Y486*/+^ mice had a normal life span with normal body weight similar to wt mice (Sup. Figure 1 A-B).

In addition, we also checked the expression profiles of *Dhtkd1* in different tissues from wt C57/129 mice. The real-time PCR assay showed that *Dhtkd1* is expressed widely in various organs and it is especially high in sciatic nerve, which is the largest nerve in the peripheral nervous system (Sup. Figure 2). In line with our previous in vitro study, we observed a gradual decrease of the *Dhtkd1* expression level in wt, *Dhtkd1*^Y486*/+^ and *Dhtkd1*^Y486*/Y486*^ in *Dhtkd1* abundant-expressing tissues including liver and sciatic nerve (Fig. 1g-h) [14]. Compared with wt mice, *Dhtkd1*^Y486*/+^ mice showed more than 50% reduction of *Dhtkd1* mRNA and protein expression, while *Dhtkd1*^Y486*/Y486*^ mice had very low *Dhtkd1* mRNA and protein expression (Fig. 1g-h). Additionally, *Dhtkd1* expression in the heterozygous ES cells was over 50% reduced (Fig. 1d). As a consequence, the *Dhtkd1*^Y486*^ knock-in mouse model mimicks the NMD phenomenon previously reported in patients that displayed an approximately 50% reduction in DHTKD1 [9].

### *Dhtkd1*^Y486*^ mice exhibit an axonopathy, but don’t show locomotor defects

**Fig. 2:**
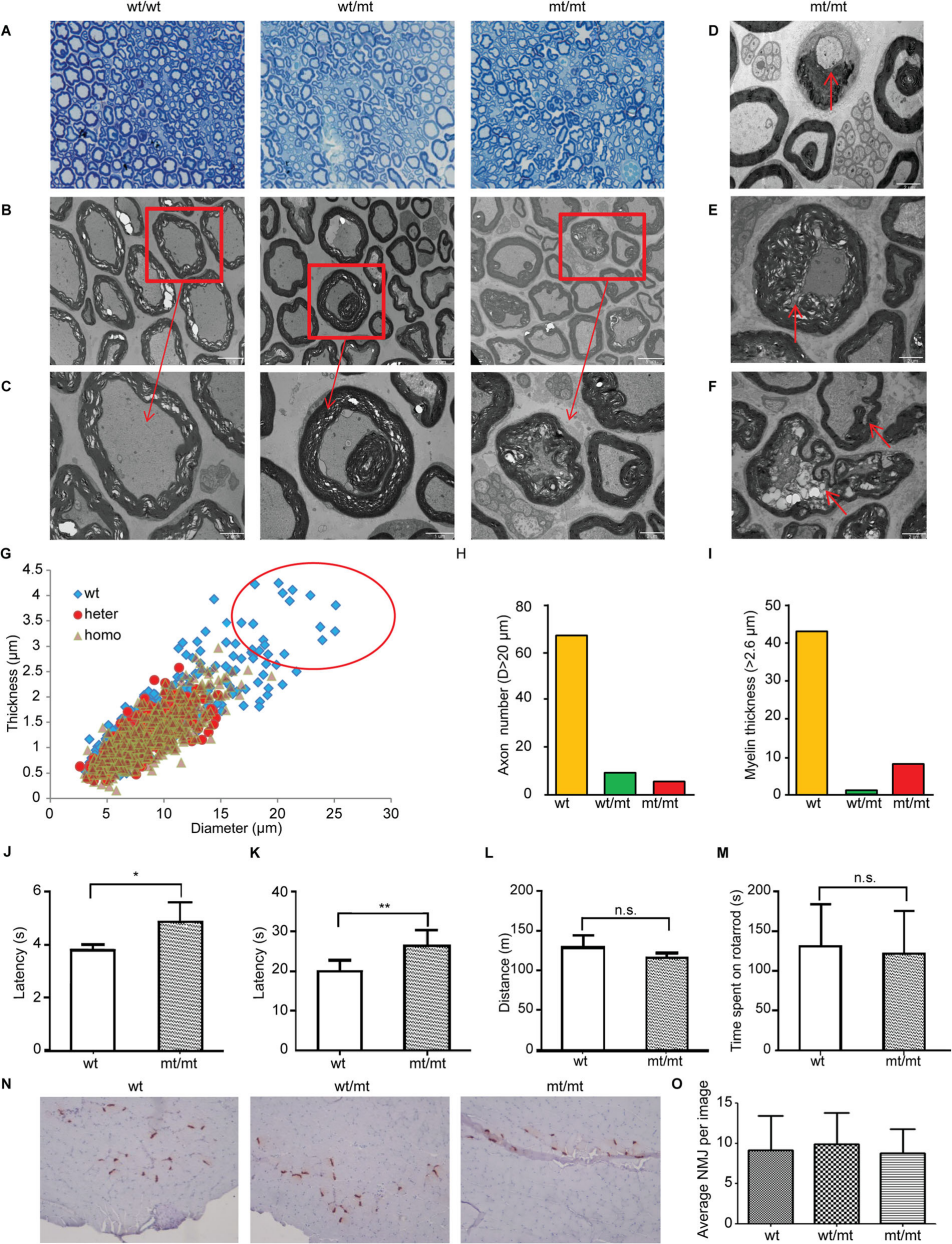
*Dhtkd1*^Y486*^mutant mice exhibited an axonopathy, but no motor defects. **a** Semi-thin sections of distal sciatic nerve from 8 months old mice were stained with toluidine blue. Images were taken at 1000x magnification. **b**, **c** Sections of distal sciatic nerve from 8 month old mice were analyzed using transmission electron microscopy (TEM). Normal and pathological changes of myelin infoldings and axon dissolutions are indicated with arrows in the wt/mt and mt/mt mice. Scale bars: 5 μm (b) or 2 μm (c) **c**. **d**-**f** Pathological changes of myelin infoldings are indicated with arrows in mt/mt mice. Scale bars: 2 μm. **g** The number of large diameter axons (> 20 μm) and myelin thickness was quantified in the wt, wt/mt and mt/mt mice. A significant difference was observed in wt/mt and mt/mt mice in comparison to the wt group. **h**, **i** The quantification of axon number (diameter > 20 μm) and number of axons with myelin thickness over 2.6 μm in distal sciatic nerve of mice. Ten randomly selected fields (50~100 nerve fibers per field) from 3 to 5 ultrathin cross sections of sciatic nerve per mouse were analyzed, and 3 mice were analyzed per genotype. **j** The sensitivity to heat of 8 months old wt and mt/mt mice was recorded using the paw pain test (*n* = 10; * *p* < 0.05). Mt/mt mice displayed a greater latency in retracting their paws in comparison to wt mice. **k** The sensitivity threshold to touch of 8 months old wt and mt/mt mice was determined using the Von Frey test (*n* = 10; ** *p* < 0.01), which also showed that mt/mt mice displayed a greater latency in retracting their paws in comparison to wt mice. **l** The running distance/min (m) of 8 months old wt and mt/mt mice was recorded in the treadmill test (*n* = 10; n.s. *p* > 0.05), with no apparent difference between genotypes. **m** The time spent on Rotarod by 8 months old wt and mt/mt mice was recorded (*n* = 8; p > 0.05), with no apparent difference between genotypes. **n** The morphology of neuromuscular junctions of wt and mt/mt mice using AChE staining. Images were taken at 100x magnification. **o** Quantification of the number of neuromuscular junctions of wt and mt/mt. AChE staining was performed on frozen sections from the skeletal muscles from 8 months old mice, with no significant difference between genotypes

To identify whether *Dhtkd1* mutations lead to neuropathological hallmarks reminiscent to CMT, the distal sciatic nerve from *Dhtkd1*^Y486*/Y486*^, *Dhtkd1*^Y486*/+^ and their wt littermates were examined using transmission electron microscopy (TEM) (Fig. 2a-f). Prominent myelin infoldings and axonal degeneration were observed in the *Dhtkd1*^Y486*/Y486*^, *Dhtkd1*^Y486*/+^ mice (Fig. 2b and c). A higher degree of axonal degeneration, myelin infoldings, and ‘double’ myelination was observed in the homozygous *Dhtkd1*^Y486*/Y486*^ mice (Fig. 2d-f). Interestingly, TEM revealed a reduction in large caliber axons in the *Dhtkd1*^Y486*/Y486*^ and *Dhtkd1*^Y486*/+^ mice, with an abundance of smaller caliber axons in comparison to wt mice (Fig. 2g). The number of axons with a large diameter (> 20 μm) and the thickness of the the myelin sheath (> 2.6 μm) in the *Dhtkd1*^Y486*/+^ and *Dhtkd1*^Y486*/Y486*^ mice were significantly reduced when compare to wt littermates at 8 months of age (Fig. 2h and i)*.*

Moreover, behavioral tests demonstrated that *Dhtkd1*^Y486*/Y486*^ mice had a significant longer latency in both the hot plate test (Fig. 2j) and the Von Frey test (Fig. 2k). The increased response latencies indicate a sensory defect, which is in line with the sensory loss in CMT2Q patients [9]. However, no difference between *Dhtkd1*^Y486*/Y486*^ mice and their respective wt littermates was detected in locomotor performance tests, including the treadmill tests (Fig. 2l) and the rotarod test (Fig. 2m). At the histopathological level, no significant difference was observed between mutant and wt mice in the number of the neuromuscular junction as was determined by acetylcholinesterase (AChE) staining (Fig. 2n and o). When the motor nerve conduction velocity (MNCV) was assessed, no significant difference was detected between the wt and mutant mice (Sup. Figure 3a-b), which would indicate an axonopathy rather than a demyelinating neuropathy. Taken together, the results suggest that alterations in the axons with secondary myelin abnormalities seen in the sciatic nerve were not sufficient to affect either MNCVs or the motor function of the knock-in mice.

### Mitochondrial accumulations in gastrocnemius muscle of Dhtkd1 deficient mice

**Fig. 3:**
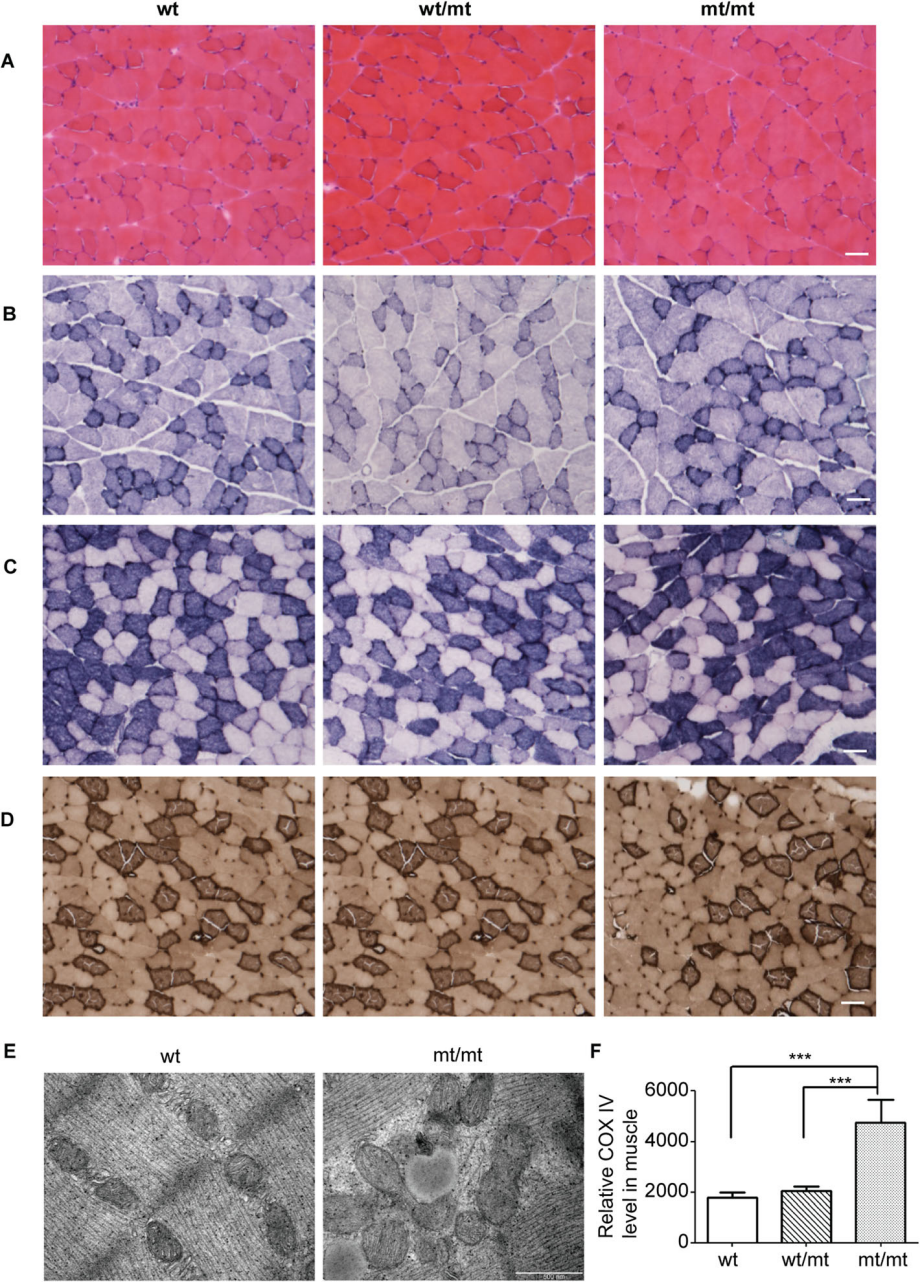
Mitochondrial accumulations in gastrocnemius muscles of mutant mice. Transverse sections (15 μm thick) from the gastrocnemius muscles of 8 month old mice and H&E staining (**a**), NADH-TR staining (**b**), SDH staining (**c**) and an ATPase staining (**d**) showed no apparent difference between genotypes. Scale bars: 50 μm. **e** Accumulation of mitochondria in the gastrocnemius muscles of mt/mt in comparison to wt mice determined by electron microscope (EM). Scale bar: 500 nm. **f** Real-time PCR was used to evaluate mtDNA copy numbers of *COX IV* (cytochrome c oxidase subunit 4) from the muscle of knock-in mice. The *COX IV* is significantly elevated in mt/mt mice in comparison to wt and wt/mt mice. *COX IV* is represented as a mitochondrial gene relative to the levels of *β-actin*, a nuclear DNA (nDNA)-encoded gene (*n* = 3, ***P<0.001)

The biceps brachii muscle biopsy of CMT2Q patients displayed abnormally small and angulated muscle fibers [9]. Based on this observation, we performed histopathology of the gastrocnemius muscle, which is one of the main muscles affected in CMT. Hematoxylin-eosin staining (H&E) showed a regular size and morphology of the myofibers in both *Dhtkd1*^Y486*/Y486*^ and *Dhtkd1*^Y486*/+^ mice at 12 months of age (Fig. 3a). Previously, we reported that silencing of DHTKD1 leads to impaired energy production, evidenced by decreased ATP, total nicotinamide adenine dinucleotide (NAD+) and NADH, and NADH levels in vitro [9]. Therefore, we performed a nicotinamide adenine dinucleotide-tetrazolium reductase (NADH-TR), succinate dehydrogenase (SDH) and adenosine triphosphatase (ATPase) staining in the gastrocnemius muscles (Fig. 3b, c, d). However, we did not observe any reduction of NADH-TR and ATPase in *Dhtkd1*^Y486*/Y486*^ and *Dhtkd1*^Y486*/+^ mice (Fig. 3b, c, d). While the normal morphology and energy status of the gastrocnemius muscles in the *Dhtkd1*^Y486*^ knock-in mouse model does not reproduce the clinical histopathological observations in CMT2Q patients, these results do align with the fact that the mice have no locomotor defects.

Interestingly, when we performed a TEM analysis for the gastrocnemius muscles in the three genotypes of mice at 12 months of age, we observed obvious mitochondrial accumulations in the *Dhtkd1*^Y486*^ knock-in mice compared to wt littermates (Fig. 3e). This is supported by the observation that the copy number of mitochondrial DNA in the *Dhtkd1*^Y486*/Y486*^ as determined by real-time PCR was significantly increased (Fig. 3f). Morphologically, mitochondria have a larger size in the *Dhtkd1*^Y486*/Y486*^ mice compared to the wt mice (Fig. 3e).

### Elevated metabolic state may be responsible for the normal locomotor behavior of *Dhtkd1*^Y486*/Y486*^ mice

**Fig. 4:**
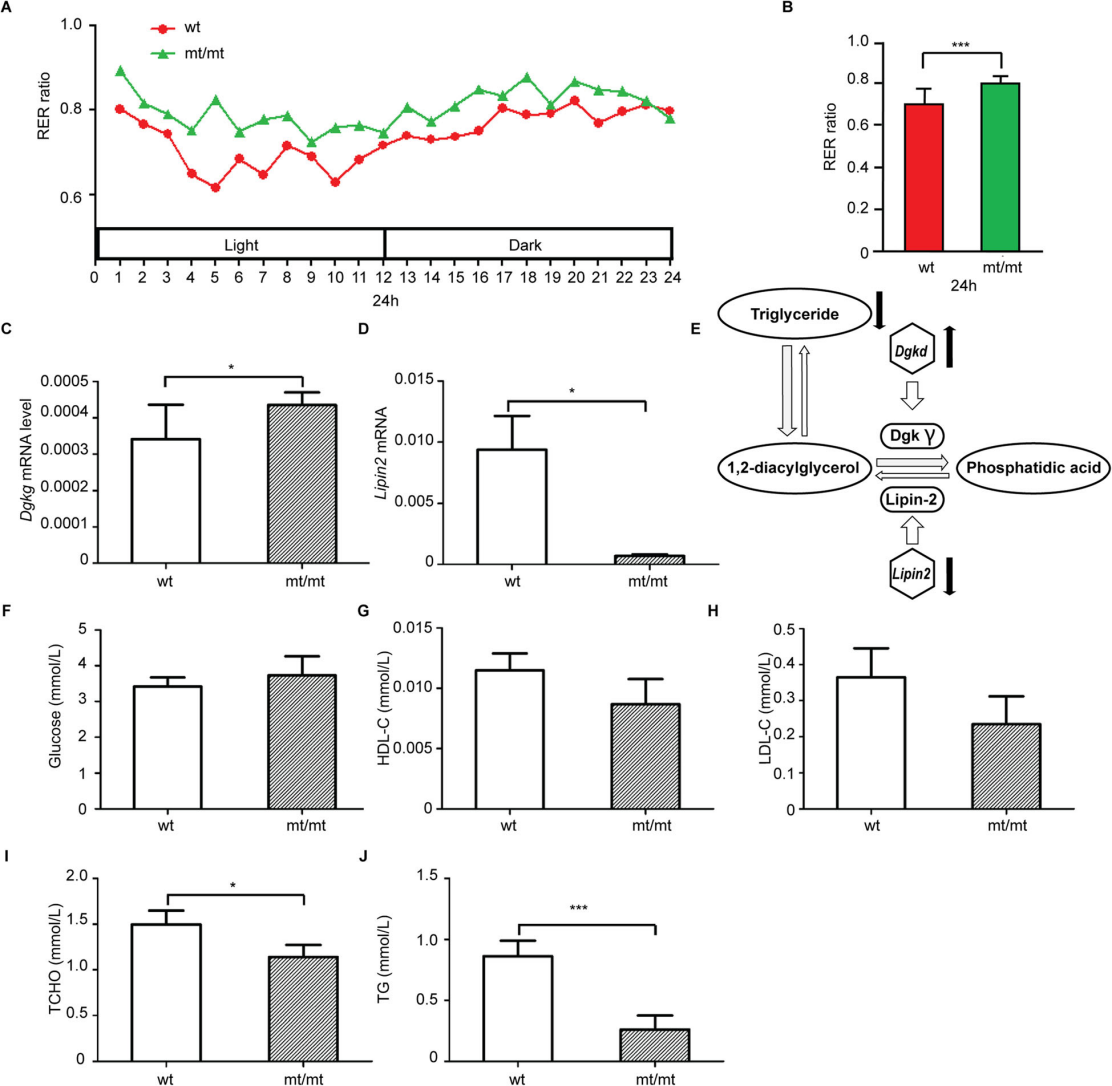
Elevated metabolic state in *Dhtkd1*^Y486*/Y486*^ mice. **a** The rate of carbon dioxide production (VCO_2_) to oxygen consumption (VO_2_) - respiratory exchange ratio (RER) - was demonstrated to be elevated in the mt/mt mice in comparison to the wt mice. The quantification of RER ratio is shown in **b**. Data are represented as the mean ± SEM of at least two independent experiments (wild type, *n* = 8; mutant, *n* = 8) over 24 h after 12 h acclimation to the metabolic chamber. Statistical significance was determined by a two-tailed Students t-test (*p < 0.05, **p < 0.01). **c** Relative level of *Dgkg* mRNA expression level determined by real-time PCR in wt and mt/mt mice. **d** Relative level of *Lipin2* mRNA expression level was shown to be significantly downregulated in mt/mt in comparison to wt mice using real-time PCR. **e** Schematic illustration showing major components regulating the triglyceride (TG) association with diacylglyceride (DAG) and phosphatidic acid (PA) interconnected pathways. No significant difference was observed between mt/mt and wt mice in terms of **f** serum glucose levels, **g** high-density lipoprotein cholesterol (HDL-C), or **h** low-density lipoprotein cholesterol (LDL-C) levels. **i** Total cholesterol (T-CHO) was significantly reduced in mt/mt versus wt mice **j**. Additionally, triglyceride (TG) levels were significantly reduced in mt/mt versus wt mice. Data are expressed as means ± SEM. Statistical significance was determined by a two-tailed Students t-test (*p < 0.05; ***p < 0.001)

In order to investigate whether energy metabolism was affected by the *Dhtkd1*^Y486*^ point mutation in vivo, we performed indirect calorimetry in *Dhtkd1*^Y486*/Y486*^ mice and their wt littermates. Oxygen consumption (VO_2_) and carbon dioxide production (VCO_2_) were measured during 24 h. This revealed that the respiratory exchange ratio (RER, VCO_2_/VO_2_) in *Dhtkd1*^Y486*/Y486*^ mice (approx. 0.8) was higher than in wt mice (approx. 0.7) continuously along 24 h (Fig. 4a and b). This indicates that the *Dhtkd1*^Y486*/Y486*^ mice have an elevated metabolic state. As DHTKD1 is presumed to be a nuclear-encoded mitochondrial precursor protein, we speculate that this elevated metabolic state in mutant mice could be a consequence of the accumulation of mitochondria [9].

Moreover, using the Affymetrix Mouse Gene 2.0 ST (Affymetrix, Santa Clara, CA) RNA chip, we could detect - at the transcriptional level - an upregulation in glycerolipid metabolic genes in the *Dhtkd1*^Y486*/Y486*^ mice in comparison to their wt littermates (Sup. Table 2). The expression of the *Dgkg* gene which encodes diacylglycerol kinase gamma protein was significantly increased in *Dhtkd1*^Y486*/Y486*^ mice (Fig. 4c). Diacylglycerol kinase can phosphorylate diacylglycerol (DAG) to phosphatidic acid (PA) [15, 16]. The expression of the gene *Lipin2*, which encodes the lipin2 protein that can turn PA into DAG, was significantly decreased in *Dhtkd1*^Y486*/Y486*^ mice (Fig. 4d). The increase of *Dgkg* and the decrease of *Lipin2* resulted mainly in the conversion of DAG. As a consequence, the levels of DAG and triglyceride (TG) are lower and the PA level are higher in blood serum in *Dhtkd1*^Y486*/Y486*^ mice (Fig. 4e). Although the glucose (GLU) level did not show a difference (Fig. 4f), and the blood lipid level of the high-density lipoprotein-cholesterol (HDL-C) and the low-density lipoprotein-cholesterol (LDL-C) showed a decreased trend (Fig. 4g and h). The level of the total cholesterol (TCHO) and triglyceride (TG) were markedly decreased in *Dhtkd1*^Y486*/Y486*^ mice compared to wt littermates (Fig. 4j and k).

## Discussion

In this study, we present a new knock-in mouse model for the *Dhtkd1*^Y486*^ mutation causing CMT2Q. This is the first time that ubiquitous expression of mutant Dhtkd1 is reported at endogenous levels in a mouse model. As the current mouse model harbors the human disease-causing mutation, it was anticipated to be an accurate modelling of the physiological and genetic condition of CMT2Q patients.

Clinically, mutations in CMT-causing genes can result in different phenotypes, ranging from normal to severely disabled, even within the same family. Therefore, it is not surprising that there might be a plethora of genetic and epigenetic factors that modify disease severity in these patients [17]. Moreover, mutations in *DHTKD1* are not an exception on this, with patients clinically presenting with a range of neurological conditions to none at all [18, 19].

Previously, we reported that the non-sense heterozygous mutation causes a reduction of approximately 50% of the DHTKD1 expression in the peripheral blood samples of CMT2Q patients [9]. The current mouse model demonstrates also a reduction of Dhtkd1 expression in the *Dhtkd1*^Y486*/+^ and *Dhtkd1*^Y486*/Y486*^ mice, which resembles that of the human CMT2Q patients. Furthermore, the *Dhtkd1*^Y486*/Y486*^ mice recapitulate the sensory defects (Fig. 2j, and k), which is a clinical phenotype reported in some CMT2Q patients [9].

The generation of the knock-in mice gives us the opportunity to investigate the pathological changes in peripheral nervous tissue, which clinically has never been done before. An axonopathy in both *Dhtkd1*^Y486*/+^ and *Dhtkd1*^Y486*/Y486*^ mice was observed, including a reduction in the number of large caliber axons (Fig. 2h and i) and the development of vacuolar inclusions at the axoplasm accompanied with myelin infoldings (Fig. 2f). Additionally, the presence of ‘double’ myelination of axons by Schwann cells was commonly observed (Fig. 2b and c). Double myelination occurs when outer myelin internodes are maintained despite losing contact with the axon [20]. This typically has been demonstrated during Wallerian degeneration, but it may also be due to retrograde inversion of infolded myelin loops, which has been previously described in a mouse model of CMT4B2 [20, 21]. Nerve conduction velocities were normal in *Dhtkd1*^Y486*/Y486*^ mice (Sup. Figure 3), despite a loss of large myelinated axons in the sciatic nerve. However, as the sciatic nerve is mainly composed of myelinated sensory neurons, this may indicate why only a sensory phenotype was detectable in adult mice (Fig. 2j and k) [22]. Therefore, mutations in *Dhtkd1* may predominant have pathological effect on the largest metabolically active sensory neurons or Schwann cells or both in our knock-in mouse model (Fig. 2g, h and i).

A possible explanation for the lack of detectable locomotor defects in these knock-in mice may be a metabolic-related compensatory mechanism that enables maintenance of normal motor functions. The physiological function of DHTKD1 in coding mitochondrial precursor protein led us to check the mitochondrial state in the gastrocnemius muscles. Interestingly, the knock-in mice showed an accumulation of mitochondria in their gastrocnemius muscles (Fig. 3e). It is known that accumulation of damaged mitochondria plays an important landmark of aging and neurodegeneration [23]. However, the accumulated mitochondria in *Dhtkd1*^Y486*/Y486*^ mice did not appear to be damaged. These mitochondria have regular substructures including clear cristae and intramembrane space (Fig. 3e). This indicates that the accumulated mitochondria are still functional. Contradictory to the decreased ATP production caused by knocking down DHTKD1 in vitro, the ATPase staining in gastrocnemius muscles of *Dhtkd1*^Y486*/Y486*^ mice did not show any differences between wt and knock-in mice, which implies that the mitochondria were functional (Fig. 3d) [9]. Combined with the fact that the expression level of Dhtkd1 was remarkably decreased in knock-in mice, we assume that increased mitochondrial biogenesis compensates for the insufficient nuclear coded mitochondrial precursors caused by the non-sense mutation. Thereby, the mitochondrial metabolic capacity remains normal and eventually supports the motor functions in our knock-in mice. This is also in line with the fact that patients with 2-aminoadipic and 2-oxoadipic aciduria related biallelic mutations in *DHTKD1* are only mildly symptomatic or are asymptomatic. As a consequence, compensatory mechanisms might also apply to human [18].

Moreover, the overall energy metabolism of *Dhtkd1*^Y486*/Y486*^ mice appears elevated in comparison to wt mice based on the indirect calorimetry assay (Fig. 4a and b). The apparent increase in metabolism may compensate for the locomotor defects in adult knock-in mice. Recently, altering the metabolic state in CMT rodent models has been targeted as a therapeutic intervention. Lipid metabolism in peripheral nerves was demonstrated to be downregulated in a rat model of CMT1A - the most common form of CMT – and using a high lipid diet, it was shown to mitigate disease pathogenesis [24]. In addition, the ketogenic diet – a high fat and low carbohydrate diet – has been shown to be beneficial in other myelinopathies, such as multiple sclerosis and Pelizaeus–Merzbacher disease [25, 26]. As a consequence, the metabolic state of peripheral tissue can strongly influence the phenotype in rodent models, as well as in patients.

In conclusion, we show that the *Dhtkd1*^Y486*^ knock-in mouse model partially recapitulates clinical phenotypes of CMT2Q patients, especially in relation to the sensory deficit and axonopathy. We hypothesize that the lack of locomotor defects might be due to a mechanism that compensates for the defects in energy metabolism. Lastly, as altered metabolic states in peripheral neuropathies are becoming more apparent as a cause or consequence of the genetic mutation, targeting metabolism through dietary changes may be beneficial for the current mouse model and possibly also for patients.

## Materials and methods

### Construction of *Dhtkd1*^Y486*^ knock-in targeting vector

The *Dhtkd1* targeting vector was obtained by the Red/ET cloning method [16]. Briefly, homology arms (A, B, C, D) were amplified from a BAC (Bacterial Artificial Chromosome) clone containing the *Dhtkd1* genomic sequence. Primers were as follows:

A1: TTTAAGCTTGGCTGTGTTCACTCATCACATT, and

A2: ATGCTCGAGTGCTACTGTTTCGTGGTACCTT,

B1: ATGCTCGAGGTGCAGAGGAAGCCAACACT, and

B2: ACAAGGATCCTCTTTACCTCCCTGGTGCTG,

C1: TTTCCGCGGAAAGCAGTTGGGTGCTCTTACC, and

C2: ACAAGGATCCTAGGTGACCCCTGTGAAAAGGT,

D1: TTTAAGCTTACAAACATGAAGCAGCAACAGA, and

D2: TACGGGTACCCTGGCATCCTCACAGACATACA.

PCR products A and B were digested by *Hind*III /*Xho*I and *Xho*I/*BamHI*, respectively, and inserted into *Hind*III *Bam*HI site of pBR322-MK-MCS to produce the retrieving vector pBR322-MK-MCS-AB. After linearization by *Xho*I, genomic DNA containing *Dhtkd1* was retrieved from the BAC clone and inserted into the vector. A site-directed mutagenesis procedure was used to introduce the *Dhtkd1*^Y486*^ point mutation into the vector by using two partly complimentary oligonucleotides with the following sequence:

*Dhtkd1* mut-1: CATTTAACTTGGTCTAGTAGGAGGTTTTTATGTCGG;

*Dhtkd1* mut-2: AACCTCCTACTAGACCAAGTTAAATGACCACTTGG.

The resulting mutated DNA fragment was verified by Sanger sequencing. Similarly, PCR products C and D were digested by *Sac*II/*Bam*HI and *Hind*III/*Kpn* I, respectively, and ligated to *Sac* II/*Bam*H I and *Hind* III/*Kpn* I sites of the PL451 vector containing the PGK-neo cassette. C-neo-D fragment was amplified from the vector using primers C1 and D2, and cloned into pBR322-MK-MCS-AB to obtain the targeting vector.

### Generation of the *Dhtkd1*^Y486*^ knock-in mice

The targeting construct was linearized with *Not* I and electroporated into SCR012 embryonic stem (ES) cells. Resistant cells were selected in the presence of G418 (G418 sulfate; Geneticin; Gibco) and Ganciclovir. DNA was isolated from a total of 96 clones and analyzed by PCR to identify positive clones that had undergone homologous recombination with the targeting vector. Primers for evaluation of the 5′ arm and the 3′ arm were P1 (5′-TCATGCAGTTGGTGCGATA-3′) and P2 (5′- AGACAATCGGCTGCTCTGAT-3′); P3 (5′-TACCCGGTAGAATTTCGACGA-3′) and P4 (5′-TGGAGATGGGAGGACATACACT-3′), giving products with a length of 5040 bp and 4414 bp, respectively.

One ES cell line was injected into C57BL/6 J blastocysts, which were subsequently transferred into pseudo-pregnant females to generate chimeric offspring. Chimeras were bred with C57BL/6 J female mice to produce heterozygous mice. The homozygous mice with mutant gene were obtained by crossing between heterozygous mice. All mice were housed in a temperature-controlled facility in rooms maintained on a 12 h light/12 h dark cycle, with free access to regular chow and water.

### Genotyping of mutant mice

The genotype of the mutant mice was determined by PCR of genomic DNA extracted from mouse tail with specific primers for the WT allele (P7: 5′- CCCGTCTCTATTTTCCACCA − 3′ and P8: 5′- ATCTCTGTGGCTTTGGTGGTC − 3′) and for the targeted allele (P7 and P2). The three primers were used in a multiplex PCR with La Taq (Takara). The amplification conditions were as follows: 95 °C for 5 min and 35 cycles of 95 °C for 30 s, 58 °C for 30 s, 72 °C for 2.5 min, and a 10 min incubation at 72 °C at the end of the run. PCR products were seperated on a 1.0% agarose gel. Product length of WT and targeted allele were 1355 bp and 2462 bp, respectively.

### RT-PCR and real-time PCR

Total RNA was extracted using TRIzol Reagent (Life Technologies Inc., Gaithersburg, MD, USA) according to the manufacturer’s instructions. The first-strand cDNAs were synthesized from total RNA with oligo (dT) primers and random 9-mer primers using PrimerScript TM RTase (Takara, Dalian, China) at 37 °C for 15 min. One microliter of the reverse transcription reaction products was used as PCR template. *Dhtkd1* expression was detected by semi-quantitative RT-PCR using as primers: 5′-ATATCAGCGCCAGTTCCG-3′ and 5′-TGAGGTGCTCTGCGTAGGT-3′. Real-time PCR was carried out using SYBR® Premix Ex Taq™ (Takara) in 96-well optical reaction plates on an ABI PRISM 7900 HT Real-time PCR system according to the manufacturer’s protocol. The cycle threshold (CT) values of *Dhtkd1* primers were compared with those of *Gapdh*-specific primers using the comparative CT method. The primers used in real-time PCR were the following:

*Dhtkd1* sense, 5′-GGTGCAGCCAGAAGCATG-3′,

*Dhtkd1* anti-sense, 5′-GGAGCCCAAGGCAAGTGT-3′,

*Gapdh* sense, 5′-CCTCGTCCCGTAGACAAAATGGT-3′,

*Gapdh* anti-sense, 5′-TTGAGGTCAATGAAGGGGTCGT-3′.

### Western blot analysis

Proteins were extracted from mouse kidney and the concentration was determined using the BCA Protein Assay Kit (Beyotime, China). Equal amounts of proteins were separated by 8% SDS-PAGE and transferred to nitrocellulose membranes (catalog no. 162–0112; Whatman, UK). Membranes were blocked with 5% non-fat milk for 1 h, followed by incubation with the primary antibodies as indicated overnight at 4 °C. Antibodies used were a goat polyclonal antibody raised against Dhtkd1 (1:200 dilution; code sc-242576; Santa Cruz Biotechnology USA), a mouse monoclonal antibody against α-tubulin (1:1000 dilution; code sAT819; Beyotime, China). Infrared fluorescence on membranes was detected by using the Odyssey infrared imaging system (LI-COR Biotechnology, Nebraska, USA).

### Differentially expressed genes analysis

RNA chip from the Affymetrix Mouse Gene 2.0 ST (Affymetrix, Santa Clara, CA) was performed by GeneTech (Shanghai, China) to detect differentially expressed genes between wild-type mice and mt/mt mice. The differentially expressed genes were mapped to KEGG (http://www.genome.jp/kegg/) pathways to analyse the function of these genes.

### ATP assay

The levels of ATP were measured using an ATP assay kit (Abnova, Taiwan) according to the manufacturer’s instructions. Briefly, mouse liver and ES cells were lysed in 100 μl of ATP buffer, and then centrifuged in ice-cold buffer at 15,000 g for 2 min. Supernatant was collected and 50 μl was added to a 96-well plate. Subsequently, reaction mix (50 μl) was added to each well before incubating at room temperature for 30 min in the dark. A standard curve was generated using different ATP concentrations (0, 2, 4, 6, 8, 10 nmol/well). In the ATP colorimetric assay, O.D. was measured at 570 nm. Total ATP levels were expressed as nmol/μl.

### NADP^+^/NADPH assay

Quantification of NADP^+^/NADPH was performed using a NADP^+^/NADPH Quantification kit (BioVision, Zurich, Switzerland) according to the manufacturer’s instructions. Briefly, mice liver and ES cells were extracted with 200–400 μl NADP^+^/NADPH extraction buffer. Subsequently, the sample was centrifuged at 14,000 g for 5 min. To measure total NADP^+^/NADPH (NADPt), 50 μl of extracted samples was transferred in duplicate into a 96-well plate. Samples were heated to 60 °C for 30 min in a water bath. Subsequently, reaction mix (100 μl) was added to each well. The plates were incubated at room temperature for 5 min. Subsequently, NADPH developer (10 μl) was added to each well and the reaction was developed for 4 h. In the NADP^+^/NADPH colorimetric assay, O.D. was measured at 450 nm.

### Measurement of mtDNA copy numbers

The mtDNA copy number was detected by real-time PCR which was performed with a Mastercycler ep realplex (Eppendorf) using the SYBR Premix Ex Taq™ (Takara). The special primers were GAPDH forward and GAPDH reverse for GAPDH; and cytochrome c I up and cytochrome c I lower for cytochrome oxidase c subunit I (COI) in real-time PCR assay. A total of 25 ng of DNA was used and the number of PCR cycles to reach this amount of DNA was defined as the threshold cycle value (Ct). The copy number of mtDNA was normalized to the GAPDH content.

### Blood lipid assay

Wt mice and mt/mt mice (*n* = 12 for each) were deprived of food and water 8 h before the assay was performed. Blood sample were drawn from retrobulbar blood vessels, centrifuged at 3000 g for 10 min and the upper liquid was transferred to a new centrifuge tube and was considered as serum. Blood lipid assays were performed by Shanghai Research Centre for Model Organisms, Shanghai, China.

### Histological analysis

Sciatic nerves were fixed by transcardial perfusion with 4% paraformaldehyde, dissected and post-fixed overnight in the same fixative. Tissue was then processed for plastic embedding and transmission electron microscopy (TEM) by standard procedures. For nerve histology, 0.5 μm sections were stained with Toluidine blue and examined by light microscopy. TEM images were collected on a Jeol 1230 transmission electron microscope. Axon diameter was determined from 5 non-overlapping fields from each of three mutant and three littermate control samples. Distances were determined using the associated software. For counts of axons, left and right nerves were taken whenever possible, and counts were averaged so that each nerve represents one mouse, which is the average count of the left and right nerve. The total number of myelinated axons in each nerve was counted using light microscopy on Toluidine blue stained sections.

For muscle analysis, dissected gastrocnemius muscle were washed briefly in PBS to remove as much blood as possible, then were fixed in 10% neutral buffered formalin, embedded in paraffin blocks, sectioned (5um) transversely and stained with haematoxylin and eosin (H&E). H&E staining was performed by incubating sections in 1% formol-calcium (10 min), in Harris’ haemtoxylin (3 min; Sigma Aldrich), in water (3 min) and in eosin (3 min). For enzyme staining, the gastrocnemius muscle was dissected and frozen fresh in isopentane supercooled by liquid nitrogen. Ten-micrometer frozen transverse sections were cut with a cryostat (Leica CM 1850) AChE staining was performed by using a staining kit (DE0056) from Leagene Biotechnology (Beijing, China). The AChE incubating solution is prepared based on the protocol of the kit and the incubation time is less than 6 h in dark till grey color appear. The AChE activities are recognized based on the red to grey staining spots. Staining for Nicotinamide adenine dinucleotide (NADH), succinate dehydrogenase (SDH) and myosin ATPase (PH 4.3) using standard methods. For NADH staining, the procedures are: 1. Muscle tissue placed in a staining tray in a damp atmosphere; 2. Drop a few incubating solution (contain 30 ml 0. 05 M Tris buffer, 30 mg nitro blue tetrazolium and 24 mg β-NADH, PH7.4) to fully cover the section; 3. Incubate for 30 min at 37 °C; 4. Rinse in distilled water and mount in aqueous mountant. For SDH staining, the procedures are similar as NADH, while the incubating solution contains 15 ml 0.2 M Sodium succinate, 15 ml 0.2 M phosphate buffer (pH 7.4) and 30 mg Nitro blue tetrazolium. For both NADH and SDH staining, the incubating solution contains nitro blue tetrazolium, which gives a blue product with NADH or SDH activity. For ATPase staining, the procedures are: 1. Place one coverslip for each biopsy in a separate, labeled staining jar for incubating with 4.3 ATP solution (5 ml Barbital Acetate Solution, 10 ml 0.1 N HCl and 8 ml deionized water, PH 4.3) five minutes at room temperature and pour out the solution; 2. Pour the washing solution (contain 6 ml 0.1 M Sodium Barbital, 3 ml 0.18 M Calcium Chloride and 21 ml deionized water, PH 9.4–9.7) into the staining jar for 30s-1min and pour out the solution; 3. Pour the ATP solution (contain 75 mg ATP powder, 6 ml 0.1 M Sodium Barbital, 21 ml deionized water and 3 ml 0.18 M Calcium Chloride, PH 9.4–9.7) into the staining jar for 45 min at 37 °C; 4. Wash the staining jar with three changes of 1% Calcium Chloride for three rinses, 3 min each; 5. Wash the staining jar with three changes of 2% Cobalt Chloride for three rinses, 3 min each. 6. Add 1% ammonium sulfide to the staining jar for at least 1–3 min; 7. Rinse in the fume hood with approximately 3–5 changes of tap water; 8. Dehydrate in ascending alcohols and clear with at least two changes of xylene; 9. Mount coverslips onto labeled glass slides with Canda Balsam. All photomicrographs were taken on a Nikon N80i microscope. Images were taken under 200x magnification.

For electron microscopy, the gastrocnemius muscles were dissected, cut into 1 mm cubes, fixed in 1% glutaraldehyde in PBS at 4 °C overnight, and further processed for staining, embedding, sectioning and post-staining. Specimens were then examined and photographed with an electron microscope (Philips CM10).

### Behavioral tests

The paw pain test and the Von Frey test were both used to measure sensory abnormalities. For paw pain test, wt and mt/mt mice were fixed in the fixator and only mice’s hind paws were exposed to the infrared heat source (30 U). The latency until the animal moved its hind paws away from the heat source was recorded. For Von Frey test, the wt and mt/mt mice stood on an elevated platform in which the surface was a wide gauge wire mesh. Von-Frey hair was inserted from below, up through the holes in the mesh, to poke the undersurface of a hind paw. At threshold, the mouse’s responds by quickly flicking its hind paw away from the hair. Mechanical withdrawal threshold was defined as the minimum gauge wire stimulus that elicited withdrawal reaction.

The treadmill test and the rotarod tests were used to measure motor performance. For the rotarod test, wt and mt/mt mice were placed on a standard rotarod apparatus. The latency for a mouse to fall off the accelerating rotarod (20 rpm) was recorded. For treadmill test, made of a continuous belt (40 cm long and 14 cm wide), operating at an adjustable speed (v = 10 m/min). A grid at the end of the belt administered an electric shock to force the mouse to run. The time spent on the grid was recorded by a counter.

### Indirect calorimetry

Wt or *Dhtkd1*^−/−^ male mice (5~6 mouth old) were housed individually in metabolic cages (TES LabMaster system) for 72 h according to the instructions of the manufacturer. After the mice adapted to the metabolic cages for 12 h, volume of food consumed, total movement distance, O_2_ consumption and CO_2_ production were continuously recorded over 24 h periods.

### Statistical analysis

Data were represented as mean ± standard deviation (SD) as indicated. Statistical significance between any 2 groups was determined by a 2-tailed Student t test. Multiple group analyses were performed by ANOVA. *p* values less than 0.05 were considered as significant.

## Supplementary information

**Additional file 1: Figure S1.** Weight curves. Weight of mice belonging to the three genotypes is shown for the male (A) and female (B) mice as a function of the age of the mice. X-axis represents month. The number of mice is 10 in three genotypes. **Figure S2.** Expression profile of *Dhtkd1* gene in adult mice. Tissue-specific expression levels of *Dhtkd1* mRNA were examined in major tissues of normal adult mice using real-time quantitative PCR. The results are from two independent experiments and each sample was analyzed in triplicate. **Figure S3.** Motor nerve conduction velocity (MNCV) and sensory nerve conduction velocity (SNCV) in wild type and gene-modified mice. (A) MNCV between WT and HOMO. (B) SNCV between WT and HOMO. **Table S1.***Dhtkd1*^Y486*^ mutation does not change Mendelian segregation ratio. *Dhtkd1* mutant homozygous mice were obtained by crossbreeding heterozygous mice. Statistical analysis included the number of wt, wt/mt and mt/mt mice. **Table S2.** Pathway analysis of differentially expressed gene.

## Data Availability

The datasets during and/or analysed during the current study available from the corresponding author on reasonable request.
