# Supplementary material for: CMT2Q-causing mutation in the Dhtkd1 gene lead to sensory defects, mitochondrial accumulation and altered metabolism in a knock-in mouse model
Source: Acta Neuropathol Commun. 2020 Mar 13;8:32. doi: 10.1186/s40478-020-00901-0 (PMC7071680; doi:10.1186/s40478-020-00901-0)
Supplement: Supplementary file 1 — Additional file 1: Figure S1. Weight curves. Weight of mice belonging to the three genotypes is shown for the male (A) and female (B) mice as a function of the age of the mice. X-axis represents month. The number of mice is 10 in three genotypes. Figure S2. Expression profile of Dhtkd1 gene in adult mice. Tissue-specific expression levels of Dhtkd1 mRNA were examined in major tissues of normal adult mice using real-time quantitative PCR. The results are from two independent experiments and each sample was analyzed in triplicate. Figure S3. Motor nerve conduction velocity (MNCV) and sensory nerve conduction velocity (SNCV) in wild type and gene-modified mice. (A) MNCV between WT and HOMO. (B) SNCV between WT and HOMO. Table S1.Dhtkd1Y486* mutation does not change Mendelian segregation ratio. Dhtkd1 mutant homozygous mice were obtained by crossbreeding heterozygous mice. Statistical analysis included the number of wt, wt/mt and mt/mt mice. Table S2. Pathway analysis of differentially expressed gene. [file 40478_2020_901_MOESM1_ESM.pdf]

S. Figure1

A

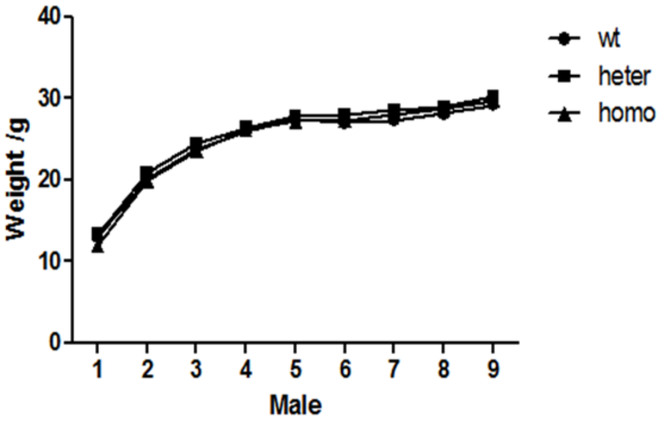
40
30
20
10
0
Weight /g
1
2
3
4
5
6
7
8
9
Male
wt
heter
homo

B

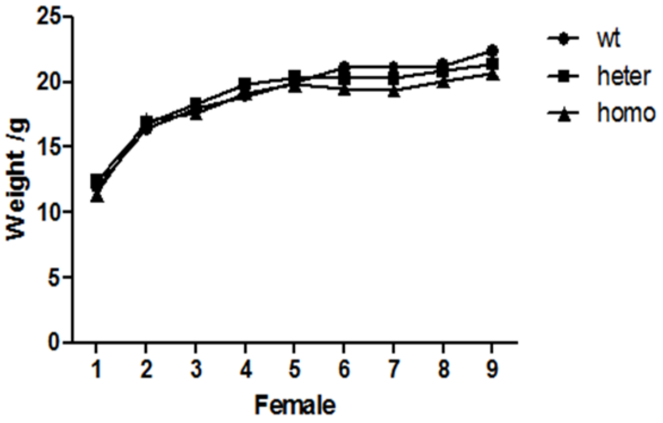
25
20
15
10
5
0
Weight /g
1
2
3
4
5
6
7
8
9
Female
wt
heter
homo

S. Figure2

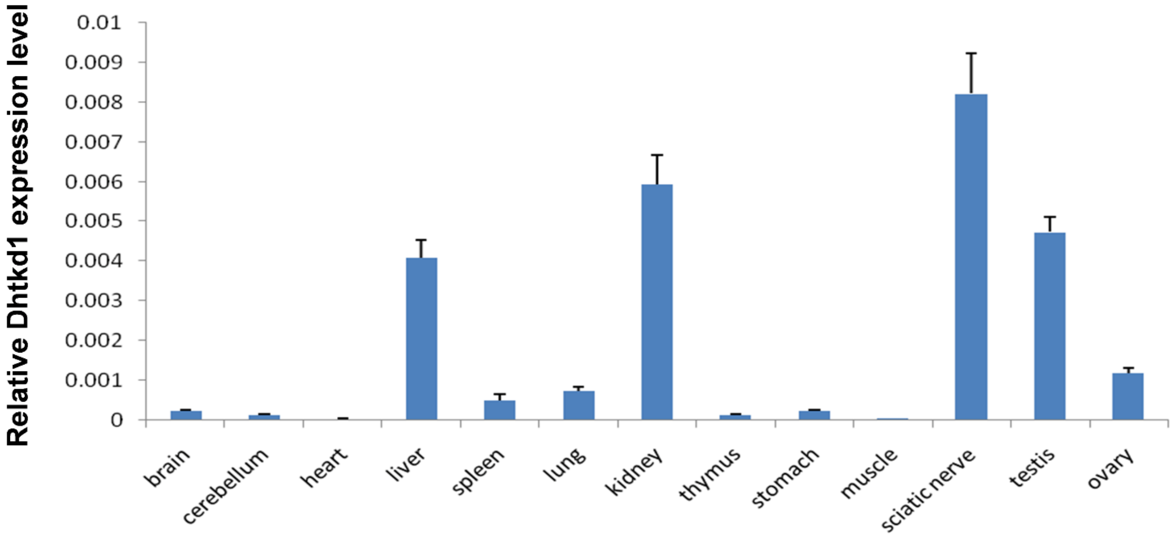

Relative Dhtkd1 expression level
0.01
0.009
0.008
0.007
0.006
0.005
0.004
0.003
0.002
0.001
0
brain
cerebellum
heart
liver
spleen
lung
kidney
thymus
stomach
muscle
sciatic nerve
testis
ovary

S. Figure 3

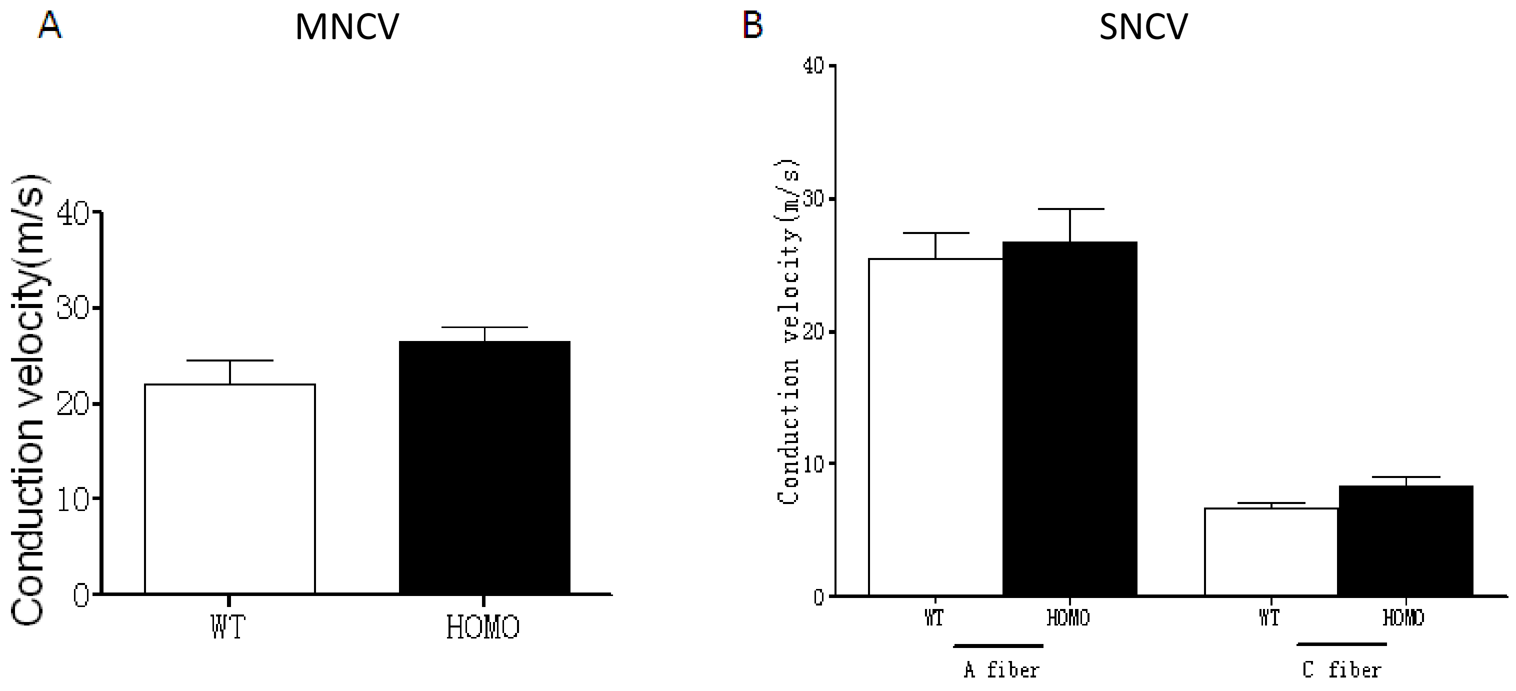
A
MNCV
Conduction velocity(m/s)
40
30
20
10
0
WT
HOMO
B
SNCV
Conduction velocity(m/s)
40
30
20
10
0
WT
HOMO
WT
HOMO
A fiber
C fiber

S. Table 1

	wt/wt	wt/mt	mt/mt	Total
Number	56	134	65	255
Percent (%)	22.0	52.5	25.5	100.0
Ratio	0.88	2.12	1.00	≈1:2:1

S. Table 2

Pathway ID	Definition	Fisher-P value	Selection Counts	Count	Enrichment Score	Genes
mmu04961	Endocrine and other factor-regulated calcium reabsorption	0.00646	3	56	2.1892	CALB1//KLK1//KLK1B22
mmu04710	Circadian rhythm	0.01878	2	31	1.72619	ARNTL//NPAS2
**mmu00561**	**Glycerolipid metabolism**	**0.05410**	**2**	**55**	**1.26679**	**DGKG//LPIN2**
mmu04740	Olfactory transduction	0.05508	12	1076	1.25899	OLFR10//OLFR141//OLFR417//OLFR507//OLFR519//OLFR576//OLFR577//OLFR6//OLFR671//OLFR791//OLFR824//OLFR968
mmu05203	Viral carcinogenesis	0.07410	4	233	1.13013	CDKN1A//CREB3L1//HIST1H4M//SP100
mmu03010	Ribosome	0.08905	3	155	1.05035	GM6139//RPL13//RPS9
